# Zero-shot evaluation reveals limitations of single-cell foundation models

**DOI:** 10.1186/s13059-025-03574-x

**Published:** 2025-04-18

**Authors:** Kasia Z. Kedzierska, Lorin Crawford, Ava P. Amini, Alex X. Lu

**Affiliations:** 1 University of Oxford, Oxford, UK; 2 Microsoft Research, Cambridge, MA USA

**Keywords:** Foundation models, Single-cell, Machine learning

## Abstract

Foundation models such as scGPT and Geneformer have not been rigorously evaluated in a setting where they are used without any further training (i.e., zero-shot). Understanding the performance of models in zero-shot settings is critical to applications that exclude the ability to fine-tune, such as discovery settings where labels are unknown. Our evaluation of the zero-shot performance of Geneformer and scGPT suggests that, in some cases, these models may face reliability challenges and could be outperformed by simpler methods. Our findings underscore the importance of zero-shot evaluations in development and deployment of foundation models in single-cell research.

**Supplementary information:**

The online version contains supplementary material available at 10.1186/s13059-025-03574-x.

## Background

Foundation models are machine learning models pretrained on huge amounts of data, where the aim of the pretraining is to enable models to capture universal patterns in data [1–3]. This foundational knowledge can either be used to specialize rapidly towards specific tasks with a small amount of additional training, or used zero-shot where the model’s internal representation of input data—an “embedding”—is used for downstream analysis with no further task-specific training.

Emerging research in single-cell biology has garnered great interest in foundation models, which promise to automate tasks such as cell type identification and gene expression prediction. Advances in pretraining and scaling foundation models mean that researchers are now seeking to understand how large unlabeled datasets can be used to initialize models with a general understanding of biology, and initiatives like CELLxGENE [4] are rising to this data demand. These developments have spurred a variety of proposed foundation models [5–12], all of which pretrain on large cell datasets.

Current proposed foundation models predominantly rely on fine-tuning, with limited exploration in zero-shot settings. However, evaluation standards for pretrained models in other biological domains, including protein sequences and biomedical images, argue that zero-shot evaluation is critical to understanding if pretraining develops a transferrable understanding of biology [13–16]. Indeed, recent research in protein language models has exposed numerous trivial mechanisms in which transfer learning can appear to boost performance on downstream tasks, but does not actually rely upon sophisticated learning from pretraining [17].

The significance of zero-shot evaluation is particularly pronounced in single-cell biology, where many tasks are exploratory and lack predefined labels that limit the feasibility of fine-tuning. This context underscores a growing need to focus on robust zero-shot performance in the field. Despite its criticality for potential applications, zero-shot evaluation remains infrequent among single-cell foundation models.

**Fig. 1:**
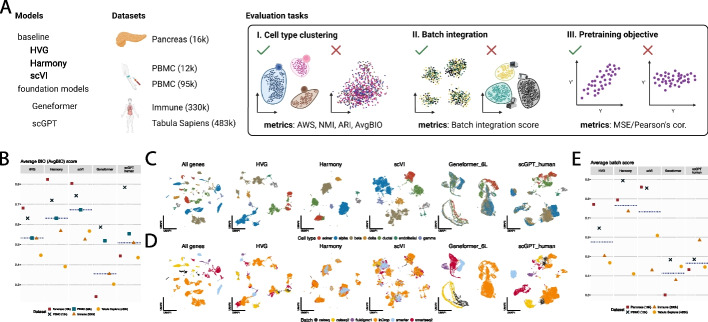
Evaluation of the cell embedding space generated by the models. **A** Overview of the evaluation setup. We compare Geneformer and scGPT to scVI, Harmony, and the selection of highly variable genes (HVG) on five diverse datasets. **B** Average BIO score for HVG and embeddings from Harmony, scVI, scGPT, and Geneformer. **C**, **D** Visualization of the UMAP projections of the Pancreas (16k) dataset using the cell embedding space generated by the models. Cells are color-coded by cell type (**C**) and batch (**D**). **E** Average batch score for HVG and embeddings from Harmony, scVI, scGPT, and Geneformer. Dashed line in **B** and **E** signifies the median calculated across the datasets

In this work, we perform zero-shot evaluations of two popular proposed single-cell foundation models, Geneformer [6] and scGPT [7] (Fig. 1A). Our evaluations are motivated by the authors’ claims that their proposed models not only generate robust cell embeddings [6] but also exhibit strong capabilities for generalizing to unseen datasets [7]. In our work, we test this claim and show that even with a set of benchmarks representing the most favorable setting where datasets consist of tissues and are generated using technologies similar to those used to pretrain these models (Additional file 2: Figs. S1 and S2), both Geneformer and scGPT underperform simpler methods. We show that zero-shot evaluation of these models exposes vulnerabilities that are not evident if evaluated with fine-tuning alone. Our results highlight the importance of zero-shot evaluation as a critical step in the development and deployment of foundation models for single-cell biology.

## Results and discussion

Both scGPT and Geneformer produce cell embeddings intended to project potentially noisy gene expression measurements to a more biologically relevant latent space [18–21], and then these cell embeddings are fine-tuned for cell type classification. However, this fine-tuning strategy fails in more exploratory contexts where cell composition in the dataset may not be known; in these settings, foundation models must produce robust cell embeddings zero-shot. We evaluated the zero-shot performance of scGPT and Geneformer in separating known cell types across multiple datasets (Fig. 1B). Both models perform worse than selecting highly variable genes (HVG) and using more established methods such as Harmony and scVI in cell type clustering, as measured by average BIO (AvgBio) score (Fig. 1B, Additional file 1: Table S1). Taking into account only the average silhouette width (ASW) metric, the established baselines still outperform scGPT and Geneformer (Additional file 2: Fig. S3, Additional file 1: Table S1). scGPT’s performance is better on the PBMC (12k) dataset compared to scVI, Harmony, and HVG; but it is worse than both scVI and Harmony with respect to AvgBIO score on other datasets (Fig. 1B, Additional file 1: Table S1). scGPT is comparable to scVI on the Tabula Sapiens, Pancreas, and PBMC (12k) datasets and outperforms Harmony on the Tabula Sapiens datasets with respect to ASW score (Additional file 2: Fig. S3, Additional file 1: Table S1). Notably, HVG outperforms Geneformer and scGPT across all metrics (Additional file 2: Fig. S4, Additional file 1: Tables S1 and S2).

Given variable performance across datasets, we sought to understand whether this variability could be explained by potential overlap between the datasets used for evaluation versus for pretraining. We find a partial overlap between the Pancreas dataset and the pretraining sets used for Geneformer. Additionally, the Tabula Sapiens and Immune datasets were included in the scGPT pretraining dataset (Additional file 1: Table S3). However, scGPT and Geneformer do not consistently outperform baselines on datasets already seen during pretraining, and the only dataset not seen during pretraining where scGPT outperforms both baselines is the the PBMC 12k study.

Next, to evaluate the impact of the pretraining dataset on zero-shot performance in cell type clustering, we tested four different variants of scGPT: randomly initialized scGPT, and scGPT pretrained on 814,000 kidney cells (scGPT kidney), on 10.3 million blood and bone marrow cells (scGPT blood), and on 33 million non-cancerous human cells (scGPT human) (Additional file 2: Fig. S5). We note that the smaller models are trained on tissue-specific data, confounding if differences in performance are due to size or the composition of dataset. However, at a minimum, scGPT human includes data used to pretrain scGPT blood and scGPT kidney. Our analysis indicates that pretraining provides a clear improvement in cell-type clustering on the PBMC (12k) dataset, and that the median score, calculated across datasets, for the three scGPT models is greater than that of the random baseline (Additional file 2: Fig. S5, Additional file 1: Table S1). We also observe that scGPT human and blood improve over random models for at least some datasets where scGPT kidney fails to, including the Immune and Tabula Sapiens datasets, suggesting that performance may improve with larger pretraining datasets. Surprisingly, scGPT human slightly underperforms scGPT blood, even for datasets involving tissue types beyond blood and bone marrow cells (Additional file 2: Fig. S5, Additional file 1: Table S4).

Overall, our findings demonstrate that scGPT and Geneformer in zero-shot configurations perform inconsistently compared to cell embeddings derived from HVG or generated using scVI or Harmony. Evaluating variants of the scGPT model highlights that while pretraining confers some benefit, beyond a certain limit, larger and more diverse datasets may no longer confer additional benefits. Additionally, models did not perform well on datasets seen during pretraining, indicating an unclear relationship between the pretraining objective and cell type clustering.

We next evaluated the zero-shot capabilities of these proposed single-cell foundation models in batch integration (Fig. 1C–E), a common task in single-cell analysis where the goal is to eliminate batch effects from multiple data sources without removing meaningful biological differences [22–24]. We first visualized the embeddings from scGPT, Geneformer, and the other baselines on the Pancreas benchmark dataset, which includes data from five different sources [25] (Fig. 1C–D). While Geneformer and scGPT-human can integrate different experiments conducted with the same experimental technique, they generally fail to correct for batch effects between techniques. Qualitatively, Geneformer’s cell embedding space fails to retain information about cell type, and any clustering is primarily driven by batch effects. While scGPT’s cell embedding space offers some separation between cell types, the primary structure in the dimensionality reduction is still driven by batch effects (Fig. 1C–D). Harmony and scVI largely succeed in integrating the Pancreas dataset.

Quantitative evaluation with batch integration metrics revealed that Geneformer underperforms relative to scGPT, Harmony, scVI, and HVG across most datasets (Fig. 1E). scVI and Harmony both outperform scGPT in datasets where the batch is restricted to technical variation (Pancreas and PBMC), but they each are outperformed by scGPT on more complex datasets where both technical and biological (i.e., variation between donors) batch effects are present (Tabula Sapiens and Immune datasets, respectively; Fig. 1E, Additional file 2: Fig. S6). We note that both the Immune and Tabula Sapiens datasets were used in pretraining scGPT, underscoring a limitation of our evaluation—we cannot disentangle if these improvements are potentially because these datasets were seen in pretraining. Although the evaluation scores for batch mixing vary—with Harmony ranking last for batch integration but second for principal component regression (PCR) score, which is expected given that Harmony adjusts the PC embeddings to correct batch effects—Geneformer consistently ranks at the bottom across all metrics (Additional file 1: Table S2).

The Tabula Sapiens dataset poses significant challenges for Harmony, particularly in terms of PCR scores, while the Immune datasets present similar difficulties for scVI (Additional file 2: Fig. S6B). Geneformer’s embeddings across all datasets show a higher proportion of variance explained by batch effects compared to the original data, indicating inadequate batch mixing (Additional file 2: Fig. S6B). Consequently, Geneformer consistently ranks last in terms of batch mixing scores (Additional file 1: Table S2), highlighting its limitations in effectively handling batch effects compared to other models.

Surprisingly, the best batch integration scores for all datasets were achieved by selecting HVG (Additional file 1: Table S2). This observation is slightly different than our qualitative observations (Fig. 1C–D) and can be explained by differences in our ranking metrics being calculated in full, rather than reduced, dimensions (Additional file 2: Fig. S6A, Additional file 1: Table S5.) However, the proposed foundation models underperform compared to both the Harmony and scVI in full and reduced dimensions (Additional file 1: Tables S4 and S6).

**Fig. 2:**
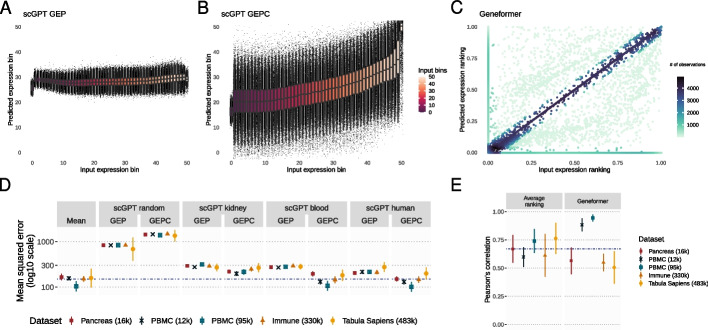
Performance comparison of scGPT and Geneformer in gene expression reconstruction. **A**–**C** Reconstruction of the expression in the Immune (330k) dataset: **A** scGPT gene expression prediction (GEP) under the masked language modeling (MLM) objective. **B** scGPT gene expression prediction from cell embeddings (GEPC). **C** Geneformer MLM output of the predicted expression ranking (y-axis) versus the true input expression ranking (x-axis). **D** Mean squared error (MSE) comparison for scGPT objectives. Mean and standard deviation range are shown as points and solid lines, respectively, and a median MSE for mean-based reconstruction is shown as a dashed line. **E** Pearson’s correlation of input and predicted expression ranking for both Geneformer and for average gene rankings. Mean and standard deviation range are shown as points and solid lines, respectively, and a median correlation for average ranking is shown as a dashed line

We propose two hypotheses as to why Geneformer and scGPT underperform zero-shot compared to the tested baselines. First, it could be that the masked language model pretraining framework used by both scGPT and Geneformer does not produce useful cell embeddings. Second, it could be that scGPT and Geneformer have failed to learn the pretraining task. To understand this distinction, we evaluated the performance of these models on the gene expression reconstruction pretraining task (for scGPT, the bin value for each gene; for Geneformer, the gene rankings) (Fig. 2, Additional file 2: Fig. S7).

We find that both models face challenges in reconstructing gene expression (Fig. 2). Without conditioning on its cell embedding, scGPT predicts the mean value of the input bin across all binned values (Fig. 2A). Predictions improve when conditioned on cell embeddings, particularly for higher input values (Fig. 2B). Under its masked language modeling (MLM) objective, Geneformer predicts the most likely gene at a given position. Although there is good agreement across many genes, there are cases in which Geneformer predicts genes absent from the input (Fig. 2C, Additional file 2: Fig. S7).

Notably, scGPT without embeddings underperforms against a naive baseline of predicting the mean, and only marginally improves with the cell embeddings (Fig. 2D). Geneformer’s gene ranking exhibits modest correlations with the actual true rankings, with median and best correlations of 0.56 and 0.95, respectively, across datasets (Fig. 2E, Additional file 2: Fig. S7).

## Conclusions

This work presents an evaluation of two single-cell foundation models, Geneformer and scGPT, in zero-shot settings. Our findings indicate that both models, in their current form, do not consistently outperform simpler baselines and face challenges in dealing with batch effects. This is in spite of these models reporting strong performance when fine-tuned, as exhibited in their original papers, demonstrating that zero-shot evaluation can reveal vulnerabilities that are not evident if models are exclusively evaluated with fine-tuning.

Our findings raise questions about the general suitability of MLM for learning single-cell embeddings. While scGPT does not outperform averaged bin prediction and struggles with accurate gene expression prediction in our evaluation datasets, Geneformer shows relative strength in blood datasets but underperforms in others. Additionally, we demonstrate that larger pretraining datasets do not always increase the performance of scGPT, and that datasets seen in pretraining still have poor cell type clustering performance. These observations suggest that improved pretraining tasks that enhance the representation of genes and gene expression within these models might be a viable path for improvement.

Recognizing the rapid advancement of the field, our study adopts a focused approach. We concentrated on specific models and selected a limited array of datasets that do not exhaustively represent all applications of single-cell analysis (for example, our datasets only include transcriptomic data). While a timely benchmark of all proposed single-cell foundation models and applications is not realistic given the fast-paced nature of the field, the aim of our work is to inform the development of future models by showing the importance of zero-shot evaluation.

In fact, some more recent advances occurring at time of our work have already sought to address limitations exposed by our analysis, for example, in improving the representation of genes by transferring knowledge from proteins [11]. However, in many cases methods do not make their pretraining code publicly available [11, 12]. Our results should be viewed as an initial exploration, highlighting specific areas for improvement and further research. We hope that our work helps shape evaluation practices in the emerging intersection of single-cell biology and foundation models.

One limitation of our analyses was that some of our evaluation datasets were used in pretraining, confounding our ability to say if performance trends are general or due to prior exposure to specific datasets. While this issue can be mitigated for individual models with strategies like identifying datasets released after a model was pretrained, this strategy may not permit a comparative analysis of older models with newer ones, as newer models may also subsume newer data for pretraining. To tackle this, we propose the creation of benchmark tasks and datasets reserved exclusively for model evaluation that should never be used to pretrain any future model, as has been done for large language models (LLMs) [26–29]. These datasets should be a representative yet separate subset of the broader available data, ensuring an unbiased and effective assessment of model performance. The curation of biologically grounded, standalone benchmark datasets would provide a substantial advancement towards more reliable and robust evaluation methods.

Building on the analyses presented in this manuscript, it would be beneficial to further explore the specific outputs provided by models like Geneformer and scGPT. Unlike traditional methods that output batch-corrected counts, Geneformer outputs rankings, and scGPT generates binned counts. Determining how effectively these models adjust for batch variations is a challenging yet an important investigation—particularly since correlating their batch correction efficacy with performance in downstream applications, such as perturbation predictions, could provide deeper insights into the practical utility of the proposed foundation models.

Another promising avenue for future research on improving these models, closely aligned with current trends in the field, is a more in-depth exploration of how they discern gene-gene interactions. Careful evaluation of this aspect is essential but requires thoughtful consideration and ideally should be paired with targeted in vitro lab experiments. These experiments are crucial for establishing a ground truth, which, in turn, enables an accurate assessment of the models’ true capabilities in unraveling complex biological interactions. A collaborative approach that combines computational evaluation with experimental validation can significantly strengthen and enhance the quality of work in designing these models.

Overall, foundation models hold significant promise for automating cell type annotation and gene expression prediction, presenting an opportunity to transform how biological data is analyzed and interpreted. Beyond technical advancements, these models have the potential to democratize scientific research by enabling groups with limited computational resources to access advanced analytical tools. The challenges identified in this study underscore the necessity of a meticulous evaluation of proposed models in zero-shot settings, ensuring that models are not only technically sound but also practically applicable. We believe that more focused evaluations, particularly in zero-shot contexts, will be instrumental to the methodological development and deployment of foundation models in single-cell research.

## Methods

### Models and baselines

We evaluated two proposed foundation models for single-cell transcriptomics: Geneformer [6] and scGPT [7]. We chose these models because they offer pretrained weights and have been trained using unsupervised objectives on extensive datasets (ca. 30M single-cell transcriptomes). Several other possible models did not have publicly available weights at the time of evaluation. Here, we provide an overview of Geneformer and scGPT, including their practices for extracting cell embeddings (i.e., latent representations of single-cells) which we follow for our analyses.

Both models accept single-cell gene expression vectors as input but represent data differently. The input to the Geneformer model is a ranked list where a gene’s position represents its expression relative to the remaining genes in the cell. The model leverages a BERT-inspired architecture with 6 Transformer layers, each with 4 attention heads. Geneformer is trained using a modification of the masked language modeling (MLM) task, where the model is trained to recover randomly selected genes that are masked or corrupted. Since genes are ordered by their expression, this effectively predicts gene expression relative to other genes. The model outputs gene embeddings, which are subsequently decoded into gene predictions. A cell embedding is calculated by averaging over all gene embeddings extracted for that cell. Genefomer was pretrained on 27.4M human single-cell transcriptomes (excluding malignant and immortalized cells).

scGPT preprocesses each gene expression vector by independently binning values into 50 equidistant bins where the lowest bin is the lowest expression and the highest bin corresponds to the highest expression. Next, the binned values and the gene tokens (i.e., a unique index for each gene) are separately embedded and summed in the embedding space—jointly representing the gene and its binned expression. Like Geneformer, scGPT uses an MLM task. However, scGPT directly learns a cell embedding, which is integrated into its pretraining loss of predicting masked genes: scGPT first predicts a masked gene expression bin and a cell embedding from unmasked genes; then, in a second step, it further iteratively refines masked gene expression using the cell embedding predicted in the first step. This means that scGPT outputs two sets of binned gene predictions in its pretraining task, first from unmasked genes alone and second from conditioning on the cell embedding. In our effort to understand the generalization of the pretraining objectives, we analyzed both. Finally, compared to Geneformer, scGPT has 3$\times$ the number of parameters, using 12 Transformer layers with 8 attention heads. scGPT is available in several variants, each pretrained on multiple different datasets. In our analyses, we focused on three variants of scGPT pretrained on 814,000 kidney cells (scGPT kidney), on 10.3 million blood and bone marrow cells (scGPT blood), and on 33 million non-cancerous human cells (scGPT human).

For baselines in evaluating cell embeddings, we compared Geneformer and scGPT against selecting highly variable genes (HVG). We standardize to 2000 HVG across all experiments. In addition, we compared all methods to scVI, a scalable generative model [20], and Harmony, a method for adjusting shared embedding space [21], which we trained on each individual dataset. While this means that we deploy scGPT and Geneformer zero-shot but train scVI and Harmony on target data, we reason this set-up reflects practical settings where resources are often more readily available to train lightweight models than to fine-tune larger ones. Importantly, both Harmony’s and scVI’s design inherently incorporates batch labels in its training process. This aspect of the baselines leverages its capability to handle batch effects directly. In contrast, Geneformer and scGPT are not explicitly pretrained with batch labels. Instead, they aim to learn to mitigate batch effects indirectly through exposure to a vast diversity of cells during their pretraining phase. For the evaluation of the pretraining objective, we used the mean estimates or average ranking as a reference.

### Datasets

To assess the quality of cell embeddings and performance on batch integration tasks, we used five distinct human tissue datasets (Additional file 1: Table S7). These datasets include samples from the pancreas [25], two sets of peripheral blood mononuclear cells (PBMCs) [30, 31], a cross-tissue immune cell atlas [32], and a multi-organ human cell atlas [33]. Each dataset poses unique challenges relevant to single-cell analysis, such as the distinction between well-defined and less well-defined cell type clusters, the integration of different technical batches within the same tissue (Additional file 2: Fig. S2), and the unification of data across multiple tissues (Additional file 2: Fig. S1). The overview of the datasets used for pretraining of the models and the datasets used in this manuscript are discussed below in Data availability.

Among the selected datasets, the Pancreas dataset partially overlapped with the data used to pretrain Geneformer (version with GEO ID: GSE84133 was included in the pretraining). We conducted evaluations using both the complete Pancreas dataset and its non-overlapping subset. The results were consistent between the two, leading us to include the entire Pancreas dataset for simplicity in this evaluation (Additional file 1: Tables S1 and S5). The Tabula Sapiens and Immune were included in the CellxGene collection [4] (May 2023 census) used for scGPT pretraining.

### Evaluation metrics

In this work, we evaluated the cell embedding space for its ability to separate known cell types correctly and to integrate different batches. For our evaluations, we largely followed the approach described in Luecken et al. [25] which included selecting the specific scores best fitting to the evaluated task (see the description below). We also evaluated the performance of the models at the pretraining task by evaluating their reconstruction accuracy.

#### Biological preservation scores

One key aspect of evaluating cell embeddings is the degree to which cell types are distinct within the embedding space. To assess this, we employ metrics based on Average Silhouette Width (ASW) [25] and Average Bio (AvgBIO) scores [7]. Briefly, ASW is computed by taking the difference of the between-cluster and within-cluster distances and dividing this by the larger of the two values. ASW is normalized to a range between 0 and 1, where 0 signifies strong within-cluster cohesion, 0.5 indicates overlapping clusters, and 1 denotes well-separated clusters. Higher ASW indicates better performance in separating clusters. AvgBIO is the arithmetic mean of three individual metrics [7]: ASW, Normalized Mutual Information (NMI), and Adjusted Rand Index (ARI). NMI and ARI are calculated based on Louvain clusters generated directly from the embedding space as described in Luecken et al. Briefly, the clustering is calculated across 20 resolutions, the resolution for which the clustering reported highest NMI metric is chosen. The clustering is then generated with the selected resolution and NMI and ARI metrics are then reported. AvgBIO is normalized to a unit scale, with higher values indicating better alignment between clusters and ground truth labels.

#### Batch mixing scores

To evaluate the extent of the mixing of the batches, we used a variation of the average silhouette score that we call batch integration score (average silhouette width score with respect to batch averaged across cell types) and the PCR score (as described in [25]). Briefly, silhouette scores are calculated for each cell type with respect to the batch label by taking only its absolute value, where a score of 0 is equivalent to absolute mixing and any deviation from 0 indicates the presence of a batch effect. To keep with the used convention, the score is then subtracted from 1, resulting in final scores on a scale between 0 and 1, where a final score of 0 suggests complete separation of the batches and strong batch effect while 1 signifies a perfect batch mixing and integration. The principal component regression (PCR) score compares the proportion of the variance that is explained by the batch variable between the original dataset and the embeddings of the model. We additionally defined a counterpart to AverageBIO score—the Average Batch Score—which is computed as the arithmetic mean between the PCR and batch integration scores.

#### Reconstructing gene expression

In pretraining, both models select a percentage of genes to mask; thus, this evaluation required selection of how many genes were masked in an input. To eliminate stochasticity in sampling and to recapitulate the maximally informative setting, we used *all* genes unmasked as input. We evaluated the models on the same datasets as in the cell embedding tasks. To evaluate the performance of scGPT in its pretraining objective, we used the mean squared error (MSE), as used by the original authors for the model’s loss [7]. To evaluate Geneformer’s performance in its pretraining objective, we measured Pearson’s correlation between the true and predicted rankings. For that, we transformed ordered outputs into scaled (from 0 to 1) rankings, where the highest expressed genes were assigned a rank of 1. Geneformer can output a sequence of up to 2048 genes and, when input is passed in batches, the model outputs the sequence of the length equal to that of the longest input. In our evaluations, we limit the output sequence to the length of the input sequence.

## Supplementary information

Additional file 1: Supplementary Tables S1-S7. Table S1: Scores for cell embeddings generated by the models. Scores calculated based on the whole dataset, as well in 10 repeats of subsampled for 10% of the dataset. Table S2: Rankings of median scores across datasets. Rankings are based on the median scores obtained from the evaluated datasets. A lower rank indicates better performance, highlighted in blue, while a higher rank signifies poorer performance, highlighted in red. Table S3: Source of the datasets and overlap with the data used for pretraining of the models. Table S4: Average ranking for the evaluated models. Rankings were calculated based on mean scores for biological integrationas well as batch mixing. A lower rank indicates better performance, highlighted in blue, while a higher rank signifies poorer performance, highlighted in red. Table S5: Scores for UMAP of the cell embeddings generated by the models. Scores calculated based on the whole dataset, as well in 10 repeats of subsampled for 10% of the dataset. Table S6: Average ranking for the UMAP embeddings of the embeddings generated by the evaluated models. Rankings were calculated based on mean scores for biological integrationas well as batch mixing. A lower rank indicates better performance, highlighted in blue, while a higher rank signifies poorer performance, highlighted in red.mean scores for biological integrationas well as batch mixing. A lower rank indicates better performance, highlighted in blue, while a higher rank signifies poorer performance, highlighted in red. Table S7: Overview of the used datasets.

Additional file 2: Supplementary Figs. S1-S9. Fig. S1: Tissue composition of the multi-tissue datasets. The tissue composition of the collections used for pretraining is depicted in the upper panel: **A** Geneformer and **B** scGPT. Below, the multi-tissue datasets utilized in evaluations are visualized: **C** Immuneand **D** Tabula Sapiens. Fig. S2: Technology distribution across datasets. Fig. S3: Proposed single-cell foundation models fail to outperform cell embeddings derived from HVG or generated using the scVI model. Average BIO score calculated on the highly variable genesof the log normalized input data and on the embeddings extracted from scVI, scGPT, and Geneformer models. Median value annotated with a dashed line. A higher score indicates better performance in separating clusters. Fig. S4: UMAP projection of the embeddings improves cell type separation for scVI. Scores used for assessing cell types separation in cell embedding space across raw and UMAP projected embeddings. **A** Average silhouette widthscore, **B** Normalized mutual informationscore, **C** Adjusted randscore and **D** Average BIOscore - an average of the other scores. The higher the score – the better the performance of the model. Fig. S5: Size of the pretraining dataset correlates with the performance at separating the cell types in cell embedding space. Average BIO score calculated on the embeddings extracted from selected variations of the scGPT models. The dashed line marks the median score across datasets. Fig. S6: UMAP projection of the embeddings results in lower batch integration scores. Scores used for assessing batch integration in cell embedding space across raw and UMAP projected embeddings. **A** Batch integrations score based on Average silhouette widthfor batch and label, and **B** Principal component regressionscore. The higher the score – the better the performance of the model. Fig. S7: Performance of the proposed foundation models with respect to reconstructed expression binor ranking. The predicted bins as a function of input bins for scGPT GEP, scGPT GEPCand the agreement between input and output rankings for Geneformerfor **A** Pancreas, **B** PBMC, **C** PBMC**D** Immune**E** Tabula Sapiensdatasets shown. Fig. S8: Pearson’s correlation of input and Geneformer’s predicted expression rankings with respect to fraction of masked input tokens. Shown here are results for Pancreasand PBMCdatasets. Fig. S9: Pearson’s correlation of input and Geneformer’s predicted expression rankings with respect to fraction of masked input tokens. Shown here are results for PBMC, Immuneand Tabula Sapiensdatasets.

Additional file 3: Review history.

## Data Availability

The Pancreas dataset [34–39] was downloaded from Figshare [40]. The PBMC (12k) dataset was accessed via the data.pbmc_dataset function from the scvi-tools [30] Python package. The PBMC (95k) data [31] was downloaded from the 10x dataset website at http://support.10xgenomics.com/single-cell/datasets. The Immune dataset was downloaded from the Cross-tissue Immune Cell Atlas website at https://www.tissueimmunecellatlas.org/ [32] and the Tabula Sapiens dataset was downloaded as the TabulaSapiens.h5ad.zip file from the Figshare Tabula Sapiens project https://figshare.com/projects/Tabula_Sapiens/100973 [41–43]. The code with data downloads and preprocessing steps is available in the GitHub repository.

## Code availability

The code used in our analyses is available at https://github.com/microsoft/zero-shot-scfoundation [44] and has also been archived on Zenodo [45]. It is released under the MIT License. In our analyses, we used version 0.1.6 (Sep 14, 2023) of the scGPT [7] code, and the version of Geneformer [6] corresponding to commit 5 d0082c (Nov 21, 2023). To compute described metrics, including those involving clustering within the embedding space, we used version 1.0.4 of the scib [25] package. Additionally, we have published a docker image at DockerHub (accessible via kzkedzierska/sc_foundation_evals) with the full environment setup.
