# Supplementary material for: Zero-shot evaluation reveals limitations of single-cell foundation models
Source: Genome Biol. 2025 Apr 18;26:101. doi: 10.1186/s13059-025-03574-x (PMC12007350; doi:10.1186/s13059-025-03574-x)
Supplement: Supplementary file 2 — Additional file 2: Supplementary Figs. S1-S9. Fig. S1: Tissue composition of the multi-tissue datasets. The tissue composition of the collections used for pretraining is depicted in the upper panel: A Geneformer and B scGPT. Below, the multi-tissue datasets utilized in evaluations are visualized: C Immuneand D Tabula Sapiens. Fig. S2: Technology distribution across datasets. Fig. S3: Proposed single-cell foundation models fail to outperform cell embeddings derived from HVG or generated using the scVI model. Average BIO score calculated on the highly variable genesof the log normalized input data and on the embeddings extracted from scVI, scGPT, and Geneformer models. Median value annotated with a dashed line. A higher score indicates better performance in separating clusters. Fig. S4: UMAP projection of the embeddings improves cell type separation for scVI. Scores used for assessing cell types separation in cell embedding space across raw and UMAP projected embeddings. A Average silhouette widthscore, B Normalized mutual informationscore, C Adjusted randscore and D Average BIOscore - an average of the other scores. The higher the score – the better the performance of the model. Fig. S5: Size of the pretraining dataset correlates with the performance at separating the cell types in cell embedding space. Average BIO score calculated on the embeddings extracted from selected variations of the scGPT models. The dashed line marks the median score across datasets. Fig. S6: UMAP projection of the embeddings results in lower batch integration scores. Scores used for assessing batch integration in cell embedding space across raw and UMAP projected embeddings. A Batch integrations score based on Average silhouette widthfor batch and label, and B Principal component regressionscore. The higher the score – the better the performance of the model. Fig. S7: Performance of the proposed foundation models with respect to reconstructed expression binor ranking. The predicted bins as [file 13059_2025_3574_MOESM2_ESM.pdf]

Supplementary Information

Description of the datasets

Tissue composition of the pretrained datasets and evaluation datasets

In their pretraining, both scGPT and Geneformer used large scale single-cell transcriptomics data. The authors of Geneformer curated a diverse compilation of studies, encompassing approximately 30 million cells from 39 distinct tissues (Additional File 2: Fig. S1A). The most comprehensive of the scGPT models, scGPT human, was trained on a collection from CellxGene, which included over 33 million cells distributed across 51 tissues, with more than a third originating from various brain regions (Additional File 2: Fig. S1B). The scGPT blood was pretrained on blood tissue, and the scGPT kidney on the kidney organ.

For our evaluations, we selected a diverse array of datasets including simpler single-tissue samples—such as the Pancreas (16k) and PBMC datasets—as well as more complex collections like the Immune (330k) and Tabula Sapiens (483k) datasets (Additional File 1: Table S7). All tissues represented in these evaluation datasets were also included in the pretraining datasets. The Immune (330k) dataset consists of immune cells from 12 organs, with over half of the cells coming from the tissues such as spleen, lung-draining lymph nodes, and small intestine (Additional File 2: Fig. S1C). The Tabula Sapiens (483k) dataset consists of cells across 24 organ tissues including lymph nodes, blood, lungs, and others (Additional File 2: Fig. S1D).

The Pancreas dataset partially overlapped with the data used for pretraining Geneformer, whereas both the Cross-tissue immune and Tabula Sapiens datasets were among the cells used to pretrain the scGPT human model (Additional File 1: Table S3). Despite the overlaps, the models struggled to outperform the scVI, Harmony, or the subselection of the highly variable genes for respective datasets.

Batch composition of the datasets

The annotation granularity and the technologies employed for dataset generation vary among the pretrained datasets. For instance, about 29% of Geneformer's pretrained data were produced using droplet technologies with unspecified further details. The remaining cells were generated using a mix of technologies, including sci-RNA-seq3 (18%), 10x 3' (21%), a broadly annotated 10x category (25%), and the remaining data were produced with technologies such as Drop-seq, DroNc-seq, and inDrop technologies. In comparison, the scGPT dataset was generated with 10x 3' protocols (67%), supplemented by sci-RNA-seq (13%) and 10x 5' protocols (16%). Other technologies used included Microwell-seq, Drop-seq, Smart-seq2, Seq-Well, BD Rhapsody Targeted mRNA, BD Rhapsody Whole Transcriptome Analysis, and various undefined single-cell sequencing technologies (Additional File 2: Fig. S2).

For the evaluation datasets, the PBMC dataset and 94% of the cells in the Tabula Sapiens dataset were generated using 10x technologies. The remainder of the Tabula Sapiens dataset was produced using the Smart-seq2 technology. The Pancreas dataset comprises of cell generated with 5 different technologies (Additional File 2: Fig. S2).

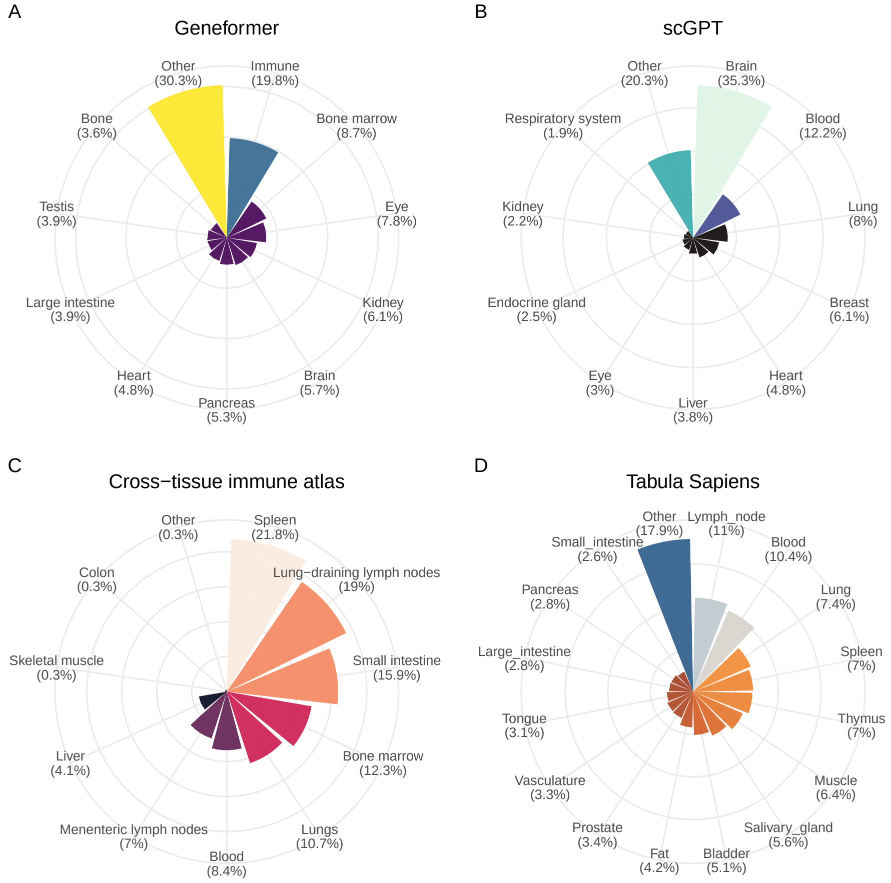

Fig. S1 Tissue composition of the multi-tissue datasets. The tissue composition of the collections used for pretraining is depicted in the upper panel: **A** Geneformer and **B** scGPT. Below, the multi-tissue datasets utilized in evaluations are visualized: **C** Immune (330k) and **D** Tabula Sapiens.

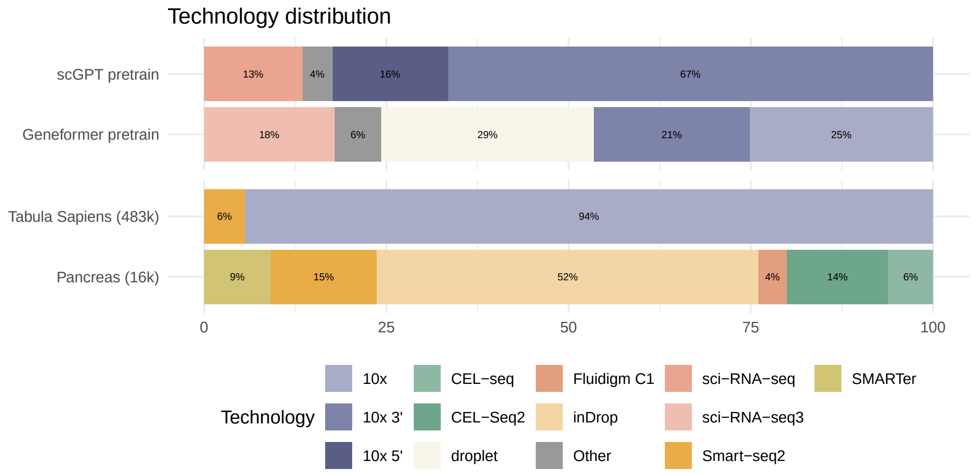

Fig. S2 Technology distribution across datasets.

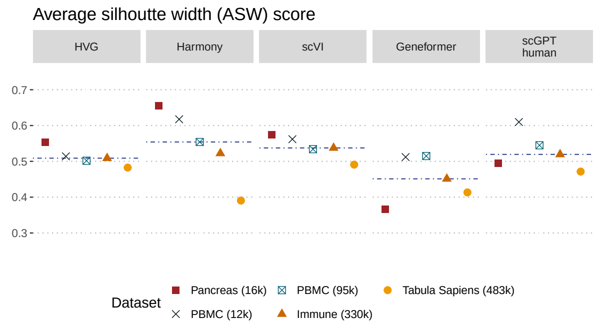

Fig. S3 Proposed single-cell foundation models fail to outperform cell embeddings derived from HVG or generated using the scVI model. Average BIO score calculated on the highly variable genes (HVG) of the log normalized input data and on the embeddings extracted from scVI, scGPT, and Geneformer models. Median value annotated with a dashed line. A higher score indicates better performance in separating clusters.

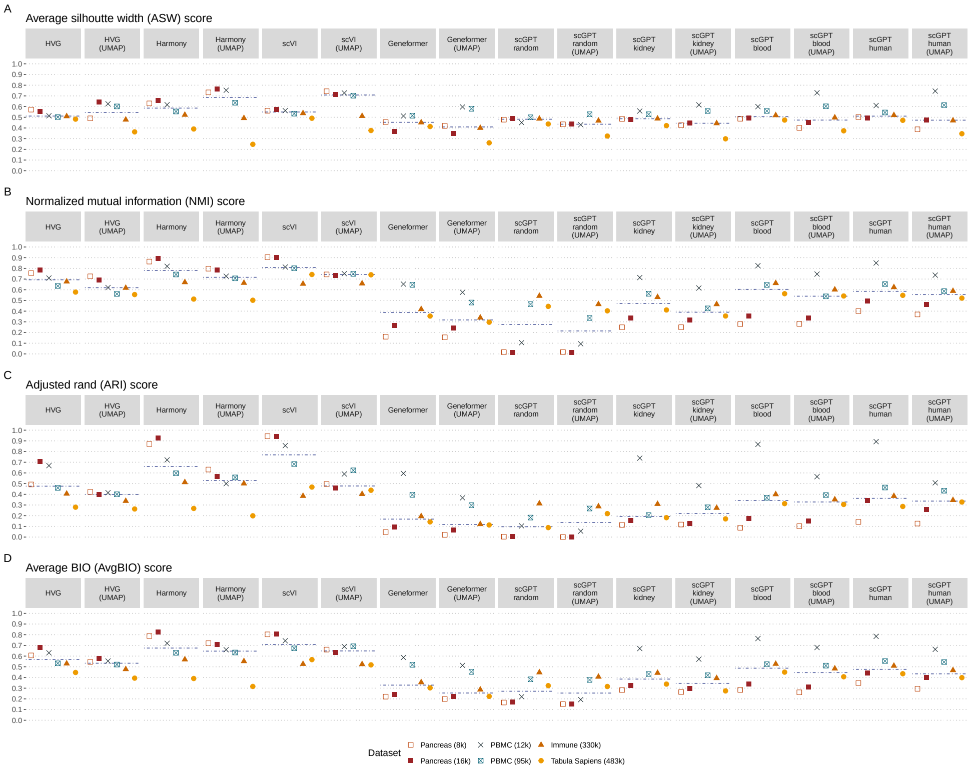

Fig. S4 UMAP projection of the embeddings improves cell type separation for scVI. Scores used for assessing cell types separation in cell embedding space across raw and UMAP projected embeddings. **A** Average silhouette width (ASW) score, **B** Normalized mutual information (NMI) score, **C** Adjusted rand (ARI) score and **D** Average BIO (AvgBIO) score - an average of the other scores. The higher the score – the better the performance of the model.

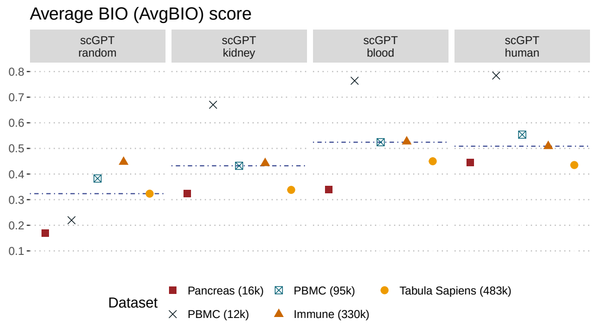

Fig. S5 Size of the pretraining dataset correlates with the performance at separating the cell types in cell embedding space. Average BIO score calculated on the embeddings extracted from selected variations of the scGPT models. The dashed line marks the median score across datasets.

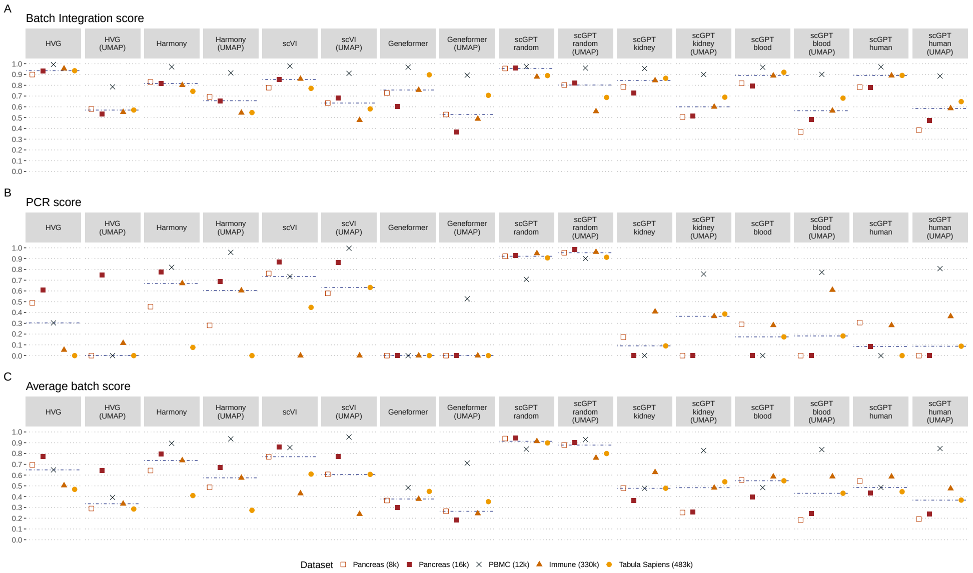

Fig. S6 UMAP projection of the embeddings results in lower batch integration scores. Scores used for assessing batch integration in cell embedding space across raw and UMAP projected embeddings. **A** Batch integrations score based on Average silhouette width (ASW) for batch and label, and **B** Principal component regression (PCR) score. The higher the score – the better the performance of the model.

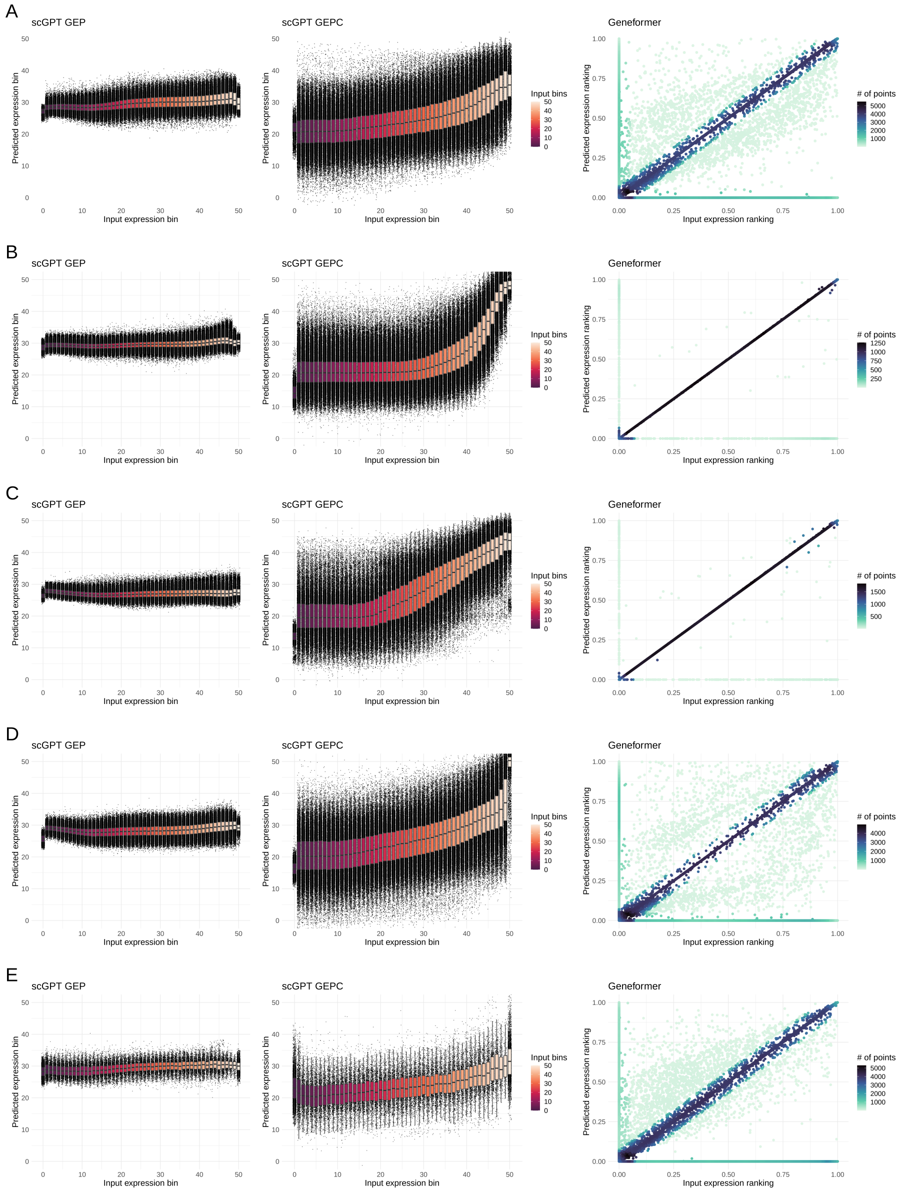

Fig. S7 Performance of the proposed foundation models with respect to reconstructed expression bin (scGPT human) or ranking (Geneformer). The predicted bins as a function of input bins for scGPT GEP (masked language modeling objective, left panel), scGPT GEPC (conditional on cell embedding, middle panel) and the agreement between input and output rankings for Geneformer (right panel) for **A** Pancreas, **B** PBMC (12k), **C** PBMC (95k) **D** Immune (330k) **E** Tabula Sapiens (483k) datasets shown.

Influence of masking on reconstruction of the expression rankings

We have run additional experiments where we provided Geneformer with a variable fraction of the masked and unmasked gene tokens. The performance of the model at the reconstruction is decreasing with the fraction of the unmasked genes (Additional File 2: Figs. S8 and S9).

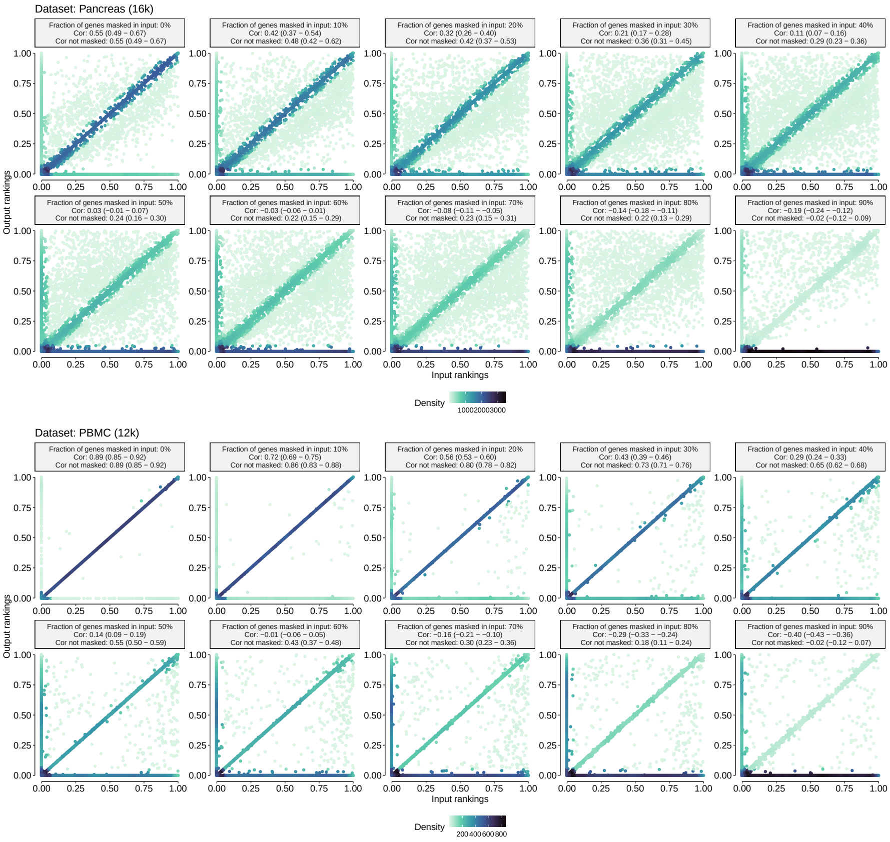

Fig. S8 Pearson's correlation of input and Geneformer's predicted expression rankings with respect to fraction of masked input tokens. Shown here are results for Pancreas (16k) (**upper panel**) and PBMC (12k) (**lower panel**) datasets.

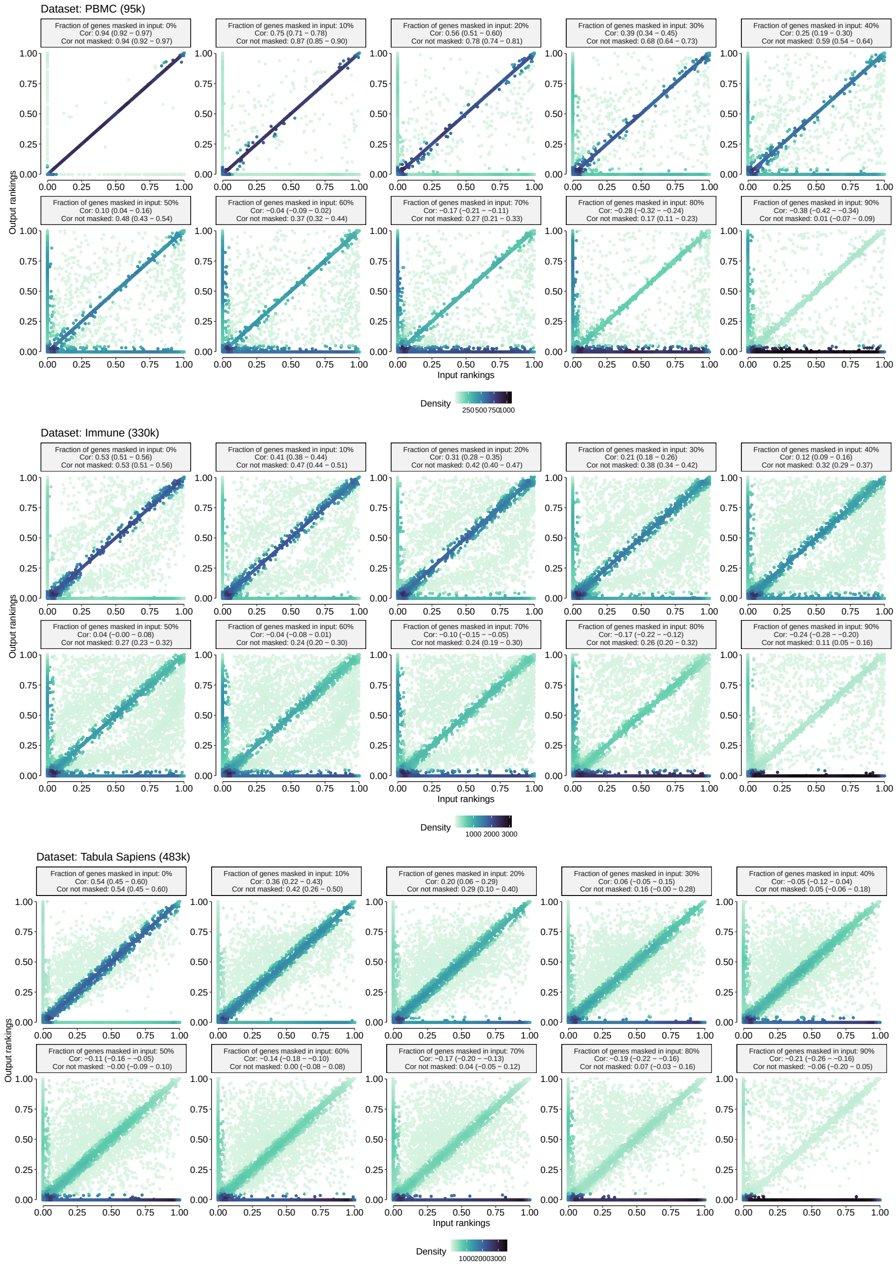

Fig. S9 Pearson's correlation of input and Geneformer's predicted expression rankings with respect to fraction of masked input tokens. Shown here are results for PBMC (95k) (**upper panel**), Immune (330k) (**middle panel**) and Tabula Sapiens (483k) (**lower panel**) datasets.
