# Supplementary material for: Zero-shot evaluation reveals limitations of single-cell foundation models
Source: Genome Biol. 2025 Apr 18;26:101. doi: 10.1186/s13059-025-03574-x (PMC12007350; doi:10.1186/s13059-025-03574-x)
Supplement: Supplementary file 3 — Additional file 3: Review history. [file 13059_2025_3574_MOESM3_ESM.docx]

Review History

**First round of review**

**Reviewer 1**

**Were you able to assess all statistics in the manuscript, including the appropriateness of statistical tests used?**

Yes, and I have assessed the statistics in my report.

**Comments to author:**

"Zero-shot evaluation reveals important limitations of foundation models in single-cell biology"

The authors present a comprehensive evaluation of foundation models Geneformer and scGPT, without fine-tuning, in embedding and clustering, batch integration, and gene expression reconstruction.

The manuscript is in good shape overall, but I have the following suggestions and questions:

- Although all scripts were provided on GitHub, the analysis details and parameters were not sufficiently described in the manuscript.

For example: How was each dataset pre-processed, and what were the QC criteria? What package/algorithm was used for HVG selection, what version, and under what settings? What package was used to calculate clustering/integration scores?

- The manuscript lacks details about the clustering/integration evaluation and the steps authors took to ensure its robustness and reliability.

Was the same clustering resolution used for all methods, or was the number of clusters fixed to the number of cell types?

If the clustering resolution was optimized for each method, with what package/algorithm and based on what metric?

- The authors state that Geneformer results on the entire Pancreas dataset and its non-overlapping subset are consistent; however, these results were not reported. I recommend including all the results in the supplementary tables.

- The caption for Figure 1 does not describe what the dashed lines represent.

- The evaluation of batch integration is based on a single metric. I recommend using other metrics from the "scib" package and reporting individual and aggregated batch integration scores, as the authors have for biological conservation metrics.

- Instead of unmasking all genes as input in evaluating gene expression reconstruction, I recommend the authors evaluate the methods with varying percentages of genes masked, and report the range of scores/errors.

- I recommend comparing to other standard batch-correction methods, for example Seurat v3.

Additionally, gene expressions can be compared to batch-corrected values from scVI and Seurat.

**Reviewer 2**

**Were you able to assess all statistics in the manuscript, including the appropriateness of statistical tests used?**

Yes, and I have assessed the statistics in my report.

**Comments to author:**

The brief communication by Kedzierska et al. aims to benchmark single-cell gene expression foundation models on two specific tasks: (1) evaluating the ability of these models to provide embeddings that reflect cell-type differences given new single-cell gene expression data, and (2) performing data integration in a way that ensures the embeddings are biological and not influenced by batch effects. However, I found the formulation of these computational questions to be simplistic and incomplete. The results presented did not convincingly demonstrate the utility or limitations of the benchmarked models.

The first set of computational experiments (Figure 1B, S1-2) seeks to answer whether the embeddings provided by scGPT or GeneFormer can distinguish cell types when given several unseen gene expression vectors. This is a complex question that needs to be addressed in more detail:

1. If the new dataset has no biological overlap with the data used to train scGPT or GeneFormer, it is unreasonable to expect sensible results. For instance, if the new dataset is from a tissue never seen in training, with a completely different set of expressed genes, clustering based on embeddings would be inapplicable. Therefore, this question should be broken down by the amount of biological overlap of the datasets.
2. It is unlikely that users will directly use the provided embeddings to cluster their data without any fine-tuning or additional analyses. Given the first point, clustering based solely on embeddings is improbable to succeed if there is no biological overlap.
3. The results presented vary significantly depending on the evaluation metric used (Figure 1B vs. S1), leading to different conclusions. This variation indicates that the current analysis is insufficient to draw solid conclusions about the utility of the embeddings.

Similarly, I was not convinced by the batch integration conclusions. Figure 1E is difficult to interpret, and the fact that scVI performs worse than HGV raises suspicions. Figure S4 demonstrates that this analysis is highly sensitive to the specific implementation, further questioning the robustness of the conclusions.

The logic behind the gene expression reconstruction experiments was also unclear. For example, it is unclear why one would expect valid reconstruction without considering the cell embedding, given that gene expression varies by cell type. The cell embedding should be integral to the reconstruction process. Perhaps I misunderstood the full details here.

A key motivation for using foundation models in the single-cell space is to learn gene-gene interactions that are cell-type or state-specific and too subtle to detect with simpler models and smaller datasets. Therefore, a thorough analysis of the learned interactions or features compared to simpler models would be a more direct and insightful approach to evaluating the merits of large foundation models.

**Authors Response**

We thank the reviewers for their comments and suggestions. We appreciate the time and effort taken to evaluate our work and are grateful for the suggestions that have helped us improve our submission.

Overview of changes

We have carefully considered each point raised and have addressed them thoroughly in the revised manuscript. Following feedback from the reviewers, we have now **(1)** added Harmony as an additional method of generating cell embeddings to our comparisons, **(2)** expanded metrics used in our evaluations by adding principal component regression (PCR) and average batch scores, **(3)** ran experiments to evaluate the influence of masking selected thresholds of the masked tokens on the model’s performance at reconstruction, **(4)** compiled a summary of the biological and technological overlap between data used in pretraining of the models and in datasets used for evaluations, **(5)** added summary rankings to ease the presentation of the results, and **(6)** clarified the necessary details of our analyses and built Docker images to improve reproducibility.

We copied the reviewers’ comments in full and listed them in heavier typeface in blue. We use a different font to denote text from the updated manuscript and we mark the revised changes in ***bold-italic***. For convenience, we also reproduced the newly added figures and tables. Below, we respond to each comment in detail:

Reviewer #1

The authors present a comprehensive evaluation of foundation models Geneformer and scGPT, without fine-tuning, in embedding and clustering, batch integration, and gene expression reconstruction. The manuscript is in good shape overall, but I have the following suggestions and questions:

- Although all scripts were provided on GitHub, the analysis details and parameters were not sufficiently described in the manuscript. For example: How was each dataset pre-processed, and what were the QC criteria? What package/algorithm was used for HVG selection, what version, and under what settings? What package was used to calculate clustering/integration scores?

- The manuscript lacks details about the clustering/integration evaluation and the steps authors took to ensure its robustness and reliability.  Was the same clustering resolution used for all methods, or was the number of clusters fixed to the number of cell types? If the clustering resolution was optimized for each method, with what package/algorithm and based on what metric?

We thank the reviewer for pointing out these important details missing from our description of our analyses. We have clarified that we use the same approach as [(Luecken et al. 2022)](https://paperpile.com/c/cT6Rz4/vIT8) implemented with the scib package. We have included all analysis details and parameters in the “Evaluation metrics” subsection of the **Methods** (see modified lines 213-229, 251-256) For convenience, we have also reproduced the relevant text below. In the “**Code availability**” section, we added the specific versions of the packages used in our evaluations. We have also specified the exact versions of packages used in our analyses in the repository and provided a Docker image allowing for reproducibility of our analyses.

The relevant amended sections read as follows (changes highlighted in ***bold-italic***):

213 **Evaluation metrics**

214 In this work, we evaluated the cell embedding space for its ability to separate known cell types correctly and to integrate different batches. ***For our evaluations, we largely followed the approach described in Luecken et al.^25^ which included selecting the specific scores best fitting to the evaluated task (see the description below).*** We also evaluated the performance of the models at the pretraining task by evaluating their reconstruction accuracy.

218 ***Biological preservation scores***

219 One key aspect of evaluating cell embeddings is the degree to which cell types are distinct within the embedding space. To assess this, we employ metrics based on Average Silhouette Width (ASW)25 and Average Bio (AvgBIO) scores^7^. Briefly, ASW is computed by taking the difference of the between-cluster and within-cluster distances and dividing this by the larger of the two values. ASW is normalized to a range between 0 and 1, where 0 signifies strong within-cluster cohesion, 0.5 indicates overlapping clusters, and 1 denotes well-separated clusters. Higher ASW indicates better performance in separating clusters. AvgBIO is the arithmetic mean of three individual metrics7: ASW, Normalized Mutual Information (NMI), and Adjusted Rand Index (ARI). ***NMI and ARI are calculated based on Louvain clusters generated directly from the embedding space as described in Luecken et al. 2022. Briefly, the clustering is calculated across 20 resolutions, the resolution for which the clustering reported highest NMI metric is chosen. The clustering is then generated with the selected resolution and NMI and ARI metrics are then reported.*** AvgBIO is normalized to a unit scale, with higher values indicating better alignment between clusters and ground truth labels.

(...)

251 Code availability

252 The code used for our analyses can be accessed at <https://github.com/microsoft/zero-shot-scfoundation>. ***Specifically, we used version 0.1.6 (Sep 14, 2023) of the scGPT7 code, and the version of Geneformer6 corresponding to commit 5d0082c (Nov 21, 2023). To compute described metrics, including those involving clustering within the embedding space, we used version 1.0.4 of the scib25 package. Additionally, we have published a docker image at DockerHub (accessible via*** [***kzkedzierska/sc_foundation_evals***](https://hub.docker.com/repository/docker/kzkedzierska/sc_foundation_evals/general)***) with the full environment setup.***

- The authors state that Geneformer results on the entire Pancreas dataset and its non-overlapping subset are consistent; however, these results were not reported. I recommend including all the results in the supplementary tables.

We thank the reviewer for this recommendation and have now included those in the amended Supplementary **Tables S6** and **S7** (referred to as S3 and S4 in the original manuscript).

*-* The caption for Figure 1 does not describe what the dashed lines represent.

We thank the reviewer for pointing this out. The description of the dashed line, which indicates the median across the datasets, has now been added to **Figure 1**. The updated caption reads as follows:

Figure 1. Evaluation of the cell embedding space generated by the models. **A** Overview of the evaluation setup. We compare Geneformer and scGPT to scVI, Harmony, and the selection of highly variable genes (HVG) on five diverse datasets **B** Average BIO score for HVG and embeddings from Harmony, scVI, scGPT, and Geneformer. **C**, **D** Visualization of the UMAP projections of the Pancreas (16k) dataset using the cell embedding space generated by the models. Cells are color-coded by cell type (**C**) and batch (**D**). E Average batch score for HVG and embeddings from Harmony, scVI, scGPT, and Geneformer. ***Dashed line in sub-panels B and E signifies the median calculated across the datasets.***

- The evaluation of batch integration is based on a single metric. I recommend using other metrics from the "scib" package and reporting individual and aggregated batch integration scores, as the authors have for biological conservation metrics.

This is a good suggestion. In the revised version of the manuscript, we have now included the principal component regression (PCR) score, which compares the change in explained variance between the original dataset and the embeddings of the model, alongside the silhouette width score with respect to the batch from the scib package. We also calculated a metric similar to the AverageBIO score, the *Average Batch Score*. The Average Batch Score represents the average across the PCR and batch integration scores. We have included the metrics in the following figures: **Fig. 1E, Fig. S8,** and the newly added **Table S3**. We outline the changes to the manuscript below.

We have also included the description of the newly included metrics in the **Methods section** (lines 230-240).

230 ***Batch mixing scores***

231 ***To evaluate the extent of the mixing of the batches, we used a variation of the average silhouette score that we call batch integration score (average silhouette width score with respect to batch averaged across cell types) and the PCR score (as described in^25^).*** Briefly, silhouette scores are calculated for each cell type with respect to the batch label by taking only its absolute value, where a score of 0 is equivalent to absolute mixing and any deviation from 0 indicates the presence of a batch effect. To keep with the used convention, the score is then subtracted from 1, resulting in final scores on a scale between 0 and 1, where a final score of 0 suggests complete separation of the batches and strong batch effect while 1 signifies a perfect batch mixing and integration. ***The principal component regression (PCR) score compares the proportion of the variance that is explained by the batch variable between the original dataset and the embeddings of the model. We additionally defined a counterpart to AverageBIO score – the Average Batch Score – which is computed as the arithmetic mean between the PCR and batch integration scores.***

In **Figure 1E**, we have now replaced the batch integration score with the *Average Batch Score*, and we have included the new score in the summary rankings (see the **new** **Table S3**).

We have also expanded the description of the batch mixing task (lines 82-86 and 88-90).

82 Quantitative evaluation with batch integration metrics revealed that Geneformer underperforms relative to scGPT, Harmony, scVI and HVG across most datasets (Fig. 1E). ***scVI and Harmony both outperform scGPT in datasets where the batch is restricted to technical variation (Pancreas and PBMC), but they each are outperformed by scGPT on more complex datasets where both technical and biological (i.e., variation between donors) batch effects are present (Tabula Sapiens and Immune datasets, respectively; Fig 1E, Fig. S8).***

(..)

88 ***Although the evaluation scores for batch mixing vary—with Harmony ranking last for batch integration but second for principal component regression (PCR) score, which is expected given that Harmony adjusts the PC embeddings to correct batch effects—Geneformer consistently ranks at the bottom across all metrics (Table S3).***

- Instead of unmasking all genes as input in evaluating gene expression reconstruction, I recommend the authors evaluate the methods with varying percentages of genes masked, and report the range of scores/errors.

We thank the reviewer for their comment. Per their recommendation, we have now run additional experiments where, at inference time, we provided Geneformer with a variable fraction of masked and unmasked values; below we include the figures for the results on the two PBMC datasets (the newly generated results for all datasets are now included in the **new Supplementary Figures S3** and **S4**). As expected, the performance of the model in predicting expression ranking decreases with an increasing fraction of unmasked genes.

In the figures below (reproduced **new** **Supplementary Figures S3** and **S4**), we show the inference-time results for two datasets, PBMC (95k) and PBMC (12k). These are the only two datasets from our original experiment where Geneformer with zero masking outperformed the average ranking baseline (**Figure 2E**). The variable-masking generation results across all other evaluation datasets are included in the **new** **Supplementary** **Figures S3** and **S4**.

Even in these best-performing datasets, the introduction of just 10% of masked tokens reduces the correlation of the output rankings to below the correlation of the average rankings (**Figure 2E**, newly generated **Supplementary** **Figures S3** and **S4**). For the PBMC (12k) dataset, we observed a reduction in correlation coefficients from 0.89 (median correlation with the 25-75% range of 0.85-0.92) for no masking to 0.72 (0.69-0.75) with 10% of tokens masked. Similarly, for the PBMC (95k) dataset, we observed a decrease in correlation coefficients from 0.94 (0.92-0.97) to 0.75 (0.71-0.78) for no masking to 10% masking, respectively. The remaining results across other evaluation datasets are included in the new **Supplementary Figures S3** and **S4** and detailed in supplementary section “***Influence of masking on reconstruction of the expression rankings*** new lines 355-358)”. Across all datasets tested, the correlation coefficient decreased quickly with an increasing percentage of masked values, dropping to an average correlation of 0.04 at 50% masking.

These results demonstrate that increasing the percentage of genes masked at inference time negatively impacts Geneformer’s performance, and that our analysis with all genes unmasked reflects the best-case scenario. Even with all genes unmasked, the Geneformer model struggles in predicting gene expression ranking across several tested datasets (new **Supplementary Figures S3** and **S4**).

We have included the following section in the Supplementary (new lines 372-375), reproduced below.

372 ***Influence of masking on reconstruction of the expression rankings***

373 ***We have run additional experiments where we provided Geneformer with a variable fraction of the masked and unmasked gene tokens. The performance of the model at the reconstruction is decreasing with the fraction of the unmasked genes (Fig. S3 and S4).***

Pearson’s correlation of input and Geneformer’s predicted expression rankings with respect to fraction of masked input tokens. Shown here are results for PBMC (12k) (**upper panel**) and PBMC (95k) (**lower panel**) datasets. Figure reproduced from **Figures S3** and **S4**.

- I recommend comparing to other standard batch-correction methods, for example Seurat v3.

Additionally, gene expressions can be compared to batch-corrected values from scVI and Seurat.

We can see what the reviewer was intending to investigate with this suggestion. Unfortunately, while we agree with the reviewer that evaluating if Geneformer and scGPT can output batch-corrected gene expression values would be interesting, these methods do not output raw counts but rather provide rankings and binned counts, respectively. This fundamental difference in data output makes a direct comparison to the batch-corrected counts from scVI, for example, complex. Benchmarking would therefore require developing a novel comparison framework, which we believe extends beyond the scope of our current study. In our manuscript ‘**Discussion**’, we have noted this as an opportunity for future work (see new lines 144-149); the relevant text is reproduced below:

144 ***Building on the analyses presented in this manuscript, it would be beneficial to further explore the specific outputs provided by models like Geneformer and scGPT. Unlike traditional methods that output batch-corrected counts, Geneformer outputs rankings, and scGPT generates binned counts. Determining how effectively these models adjust for batch variations is a challenging yet an important investigation - particularly since correlating their batch correction efficacy with performance in downstream applications, such as perturbation predictions, could provide deeper insights into the practical utility of the proposed foundation models.***

Reviewer #2

The brief communication by Kedzierska et al. aims to benchmark single-cell gene expression foundation models on two specific tasks: (1) evaluating the ability of these models to provide embeddings that reflect cell-type differences given new single-cell gene expression data, and (2) performing data integration in a way that ensures the embeddings are biological and not influenced by batch effects. However, I found the formulation of these computational questions to be simplistic and incomplete. The results presented did not convincingly demonstrate the utility or limitations of the benchmarked models.

Overall, we thank the reviewer for their thoughtful critique. We agree with the reviewer about the simplicity of our formulation. This was intentional: the aim of this work was to demonstrate that even in the most favorable settings and basic analyses, proposed single-cell foundation models do not exhibit significant performance gains. The reason why we focus on a more basic evaluation set-up is because we believe a lack of performance in these settings casts doubt on more sophisticated applications of these models. If a model cannot represent biology already seen in training, it is unlikely it can generalize to new, unseen biology. Similarly, if a model cannot represent well-resolved and distinct cell types, it is unlikely to be able to capture subtle differences across cell states. However, as the reviewer rightfully points out in their comments, we lacked clarity in how we were presenting our study and have improved this accordingly in the revised version of the manuscript.

Following the reviewer’s comments, we have now provided more information and context where needed. This includes writing changes where we explicitly clarify that all of our evaluation datasets involve tissues seen during training, provide information on how our clustering analyses relate to single-cell transcriptomic analyses, and provide clarifications on why we use a range of metrics and more holistic scores to synthesize those metrics.

The first set of computational experiments (Figure 1B, S1-2) seeks to answer whether the embeddings provided by scGPT or GeneFormer can distinguish cell types when given several unseen gene expression vectors. This is a complex question that needs to be addressed in more detail:

1. If the new dataset has no biological overlap with the data used to train scGPT or GeneFormer, it is unreasonable to expect sensible results. For instance, if the new dataset is from a tissue never seen in training, with a completely different set of expressed genes, clustering based on embeddings would be inapplicable. Therefore, this question should be broken down by the amount of biological overlap of the datasets.

We thank the reviewer for their comments.

First, consistent with our aim of demonstrating that proposed single cell foundation models fail to extract biologically informative zero-shot embeddings even in the most favorable settings, we note that all of our evaluation datasets were composed of tissues seen during pretraining (with the exception of the “scGPT kidney” and “scGPT blood” models which were trained only on the kidney and blood tissues, respectively). Outside of the Pancreas (16k) dataset, which was specifically chosen to understand robustness to sequencing technology, all evaluation datasets were primarily sequenced using 10x technologies, which form 83% of scGPT’s pretraining dataset and 46% of Geneformer’s. Considering that our evaluation datasets are all tissues seen during pretraining, and (mostly) with the same technologies, we believe this mitigates the reviewer’s concern that these models are failing because they are being deployed on biology unseen in training. We have now detailed the extent of overlap between the training and evaluation datasets in the **new** Supplementary section entitled “**Description of the Datasets**” (see new lines 343-371).

Second, we note that the reviewer’s claim that “*if the new dataset has no biological overlap with the data used to train… it is unreasonable to expect sensible results*” is a conservative one not consistent with current claims about single-cell foundation models, nor is it supported by evidence from other biological domains using foundation models. We want to be clear that our investigation was motivated by the direct claims of proposed single-cell foundation models. For instance, the authors of the scGPT model [(Cui et al. 2024)](https://paperpile.com/c/cT6Rz4/McJn) write in their Discussion:

“*We demonstrated the benefits of pretraining with comprehensive experiments in both zero-shot and fine-tuning settings. The pretrained model showcases strong capabilities of extrapolating to unseen datasets, presenting meaningful clustering patterns in accordance with cell types in zero-shot experiments.*”

We have now included this motivation in the ’**Background**‘ section of the manuscript (see new lines 30-37).

30 In this work, we perform zero-shot evaluations of two popular proposed single-cell foundation models, Geneformer^6^ and scGPT^7^ (Fig. 1A). ***Our evaluations are motivated by the authors’ claims that their proposed models not only generate robust cell embeddings^6^ but also exhibit strong capabilities for generalizing to unseen datasets^7^. In our work, we test this claim and show that even with a set of benchmarks representing the most favorable setting where datasets consist of tissues and are generated using technologies similar to those used to pretrain these models (Figs. S1 and S2), both Geneformer and scGPT underperform simpler methods.*** We show that zero-shot evaluation of these models exposes vulnerabilities that are not evident if evaluated with fine-tuning alone. Our results highlight the importance of zero-shot evaluation as a critical step in the development and deployment of foundation models for single-cell biology.

Other biological domains have also demonstrated that foundation models can generalize far beyond their training data, even when deployed zero-shot. For example, zero-shot embeddings from computer vision models trained on natural images have been shown to outperform even specialist methods for cell microscopy [(Pawlowski et al. 2016; Ando, McLean, and Berndl 2017)](https://paperpile.com/c/cT6Rz4/NU7S+cPd6). Similarly, protein sequence models trained exclusively on naturally-occurring sequences have been shown to generalize zero-shot to artificial proteins [(Hsu et al. 2022; Notin et al. 2023)](https://paperpile.com/c/cT6Rz4/DEx4+Ni5w).

Together, we hope this demonstrates that the practice of applying single-cell foundation models zero-shot to unseen data and biology is both a) currently advocated for by authors of these publications, and b) not unreasonable to expect generalization on given prior precedent for biological foundation models. In this context, we believe our contribution showing that proposed single-cell foundation models are unreliable zero-shot — even in the best-case scenario where biology is seen in pre-training — is valuable. Regardless of whether it is obvious these models should not be deployed this way, many users of these models have reason to believe these models can support general analyses on unseen data and biology, and our study provides empirical evidence against this practice.

2. It is unlikely that users will directly use the provided embeddings to cluster their data without any fine-tuning or additional analyses. Given the first point, clustering based solely on embeddings is improbable to succeed if there is no biological overlap.

Unfortunately, we have to disagree with the reviewer that it is unlikely that users will use the embeddings to cluster their data without fine-tuning. Single-cell clustering and visualization is usually the first step in a transcriptomic analysis workflow (e.g., [(Heumos et al. 2023; Slovin et al. 2021; Lun, Bach, and Marioni 2016)](https://paperpile.com/c/cT6Rz4/nkwO+raVd+8qq8)). At its core, embedding single cells with a foundation model is effectively dimensionality reduction, reducing noisy high-dimensional gene expression measurements into a more robust biological latent space. From this perspective, single-cell foundation model embeddings can be used as a drop-in replacement for more standard dimensionality reduction techniques like PCA for the purposes of single-cell clustering and visualization.

We also note that the field is already adopting zero-shot embeddings for this exact purpose. Products marketed by recent start-ups seek to facilitate this. For example, [Code Ocean](https://codeocean.com/webinars/how-everyone-can-successfully-use-generative-foundational-models-in-biotech) includes in their webinars a tutorial on how to extract embeddings from scGPT for visualization with UMAP. Open-source code produced by [helicalAI](https://github.com/helicalAI/helical?tab=readme-ov-file) has built-in functions for extracting embeddings from various proposed single-cell foundation models and includes visualization with UMAP as the [first step in their quick start tutorial](https://github.com/helicalAI/helical/blob/release/examples/notebooks/Quick-Start-Tutorial.ipynb). As in our response to the first point above, it is already routine practice in other biological data domains like protein sequences and microscopy to conduct analyses on the embeddings of biological foundation models directly, without any fine-tuning, and even on data substantially different from pretraining data, as demonstrated in many studies [(Cheng et al. 2024; Daudert 2018; Qiao et al. 2024; Israel et al. 2024)](https://paperpile.com/c/cT6Rz4/Qk2n+Hilx+853N+rTSj). Without empirical investigations like ours, there is no reason to doubt that proposed single-cell foundation models cannot be deployed similarly.

Overall, we argue that our study’s focus on clustering metrics and other behavior has practical implications, as it directly relates to one of the main workhorses in single-cell analysis. Our analysis exposes limitations in the ability of proposed single-cell foundation models to facilitate these analyses.

3. The results presented vary significantly depending on the evaluation metric used (Figure 1B vs. S1), leading to different conclusions. This variation indicates that the current analysis is insufficient to draw solid conclusions about the utility of the embeddings.

We appreciate the reviewer raising this point. While we disagree with the reviewer that variability in performance across metrics, methods, and datasets implies there are issues with our analysis (as we unpack below, this variability is the norm for single-cell benchmarking analyses, and useful conclusions can still be made in the presence of variability), we understand the reviewer’s point that focusing on one set of metrics may bias our conclusions to one particular setting. To address this second point, we have extended our analysis to include more metrics (see updated **Figs. 1E** and **S8**, **Tables S7** and **S8**), and now report average rankings across metrics and datasets (see new **Tables S3-S5**).

First, variability in performance across metrics, methods, and datasets is expected and has been previously observed in single-cell benchmarking studies (e.g., by [(Yu et al. 2022)](https://paperpile.com/c/cT6Rz4/a0ry), [(Luecken et al. 2022)](https://paperpile.com/c/cT6Rz4/vIT8), [(Luo et al. 2024)](https://paperpile.com/c/cT6Rz4/mH9W), and others). This variability can be attributed to trade-offs in performance across different settings. For example, [(Luecken et al. 2022)](https://paperpile.com/c/cT6Rz4/vIT8) report that methods that are more robust to batch effects often do not preserve nuanced biological signal. Similarly, different metrics reveal different facets of methods: some embedding and dimensionality reduction algorithms may preserve global cluster structure (as measured by e.g. ARI), at the trade-off of loss of relative distances between clusters (as measured by e.g. ASW), and this means different performance under different clustering methods (as discussed in [(Ronan, Qi, and Naegle 2016)](https://paperpile.com/c/cT6Rz4/pg75). In light of this, recent benchmarking studies have sought to report a range of metrics and datasets to capture the full spectrum of model adaptability and trade-offs between methods, and we likewise sought to replicate this standard.

However, even when datasets and clustering analysis workflows are diversified, it is still useful to report how successful methods are across different datasets on average. This is because many single-cell analyses are inherently exploratory, and prior information may not always exist to guide the selection of a method. We originally attempted to capture this in our use of the Average BIO score, which is the mean of three metrics, but we further extend this in our revision. We have now incorporated additional metrics to evaluate the mixing of the batches and created rankings to enhance the clarity of the metrics presented (newly added **Tables S3** and **S4**). While we do observe some (expected) variability in performance across these metrics, the overall rankings are consistent across individual metrics, as detailed in the new **Table S3** reproduced below.

The rankings demonstrate limited variability in ranking across the datasets used for evaluation, although scVI performs particularly poorly on the Immune (330k) dataset and Harmony performs poorly on the Tabula Sapiens (483k) dataset. Despite these challenges, these methods outperform the proposed foundation models in all datasets, as shown in the new **Table S4** (reproduced below). Notably, Geneformer consistently ranks at the bottom across all evaluations. We have included the following amendment in the “**Results”** section (lines 91-95).

91 ***The Tabula Sapiens dataset poses significant challenges for Harmony, particularly in terms of PCR scores, while the Immune datasets present similar difficulties for scVI (Fig. S8B). Geneformer’s embeddings across all datasets show a higher proportion of variance explained by batch effects compared to the original data, indicating inadequate batch mixing (Fig. S8B). Consequently, Geneformer consistently ranks last in terms of batch mixing scores (Table S3), highlighting its limitations in effectively handling batch effects compared to other models.***

Similarly, I was not convinced by the batch integration conclusions. Figure 1E is difficult to interpret, and the fact that scVI performs worse than HGV raises suspicions.

We compared our independently calculated metrics against previously published benchmarks, and observed for overlapping datasets, our values are in agreement: Luecken et al. 2022 also evaluated scVI using the same metrics as us on the Pancreas (16k) dataset, and their values are consistent with ours (PCR score in our evaluations: 0.87, in scIB paper: 0.86; Batch integration score in our evaluations: 0.85 in scIB paper: 0.85). . This suggests we are implementing these baselines in a way consistent with prior work, and thus it is unlikely there is an issue with our implementation.

In general, we do not expect our baseline methods to outperform HVG on every single dataset. Some datasets may represent settings ill-suited to the assumption of the methods. Again, this is consistent with prior work: Luecken et al. 2022 show on some datasets, unintegrated gene expression values outperform the majority of batch integration methods they benchmark. While these dataset and method-specific differences are interesting, we did not expand on them at length because the aim of our paper (by nature of being a brief communication) was not to delve into specific factors for each dataset and method. Rather, our main finding is the overall trend that Geneformer and scGPT underperform much simpler models across a range of datasets -- a finding that is surprising in the light of the claims made in the respective original Geneformer and scGPT papers. In the ‘**Results’** (lines 82-86 and 88-90), we now write:

82 Quantitative evaluation with batch integration metrics revealed that Geneformer underperforms relative to scGPT, Harmony, scVI and HVG across most datasets (Fig. 1E). ***scVI and Harmony both outperform scGPT in datasets where the batch is restricted to technical variation (Pancreas and PBMC), but they each are outperformed by scGPT on more complex datasets where both technical and biological (i.e., variation between donors) batch effects are present (Tabula Sapiens and Immune datasets, respectively; Fig 1E, Fig. S8).***

(..)

88 ***Although the evaluation scores for batch mixing vary—with Harmony ranking last for batch integration but second for principal component regression (PCR) score, which is expected given that Harmony adjusts the PC embeddings to correct batch effects—Geneformer consistently ranks at the bottom across all metrics (Table S3).***

Figure S4 demonstrates that this analysis is highly sensitive to the specific implementation, further questioning the robustness of the conclusions.

For clarification, **Figure S8** (previously Figure S4), which shows our metrics calculated directly in embedding space versus embedding space projected to two dimensions with UMAP, is intended to show performance under different workflows in single cell analysis. Even though single-cell clustering and visualization is routine in single cell analysis, how this is implemented differs from analysis to analysis. In some workflows, researchers may directly cluster the latent space, but in others, they may visualize single-cells with UMAP to form qualitative insights. Often, researchers will apply multiple workflows to inform each other (e.g., a UMAP visualization may be used to parameterize the number of clusters for a clustering method), or for a more holistic understanding of the data (e.g., [(Ronan, Qi, and Naegle 2016)](https://paperpile.com/c/cT6Rz4/pg75) argue that researchers should compare multiple clustering methods for a more robust result). Thus, we view the reviewer’s feedback here as less specific to our study, but more so highlighting the challenge that there is no single gold-standard clustering and visualization workflow for single-cell analysis in general (and as argued by [(Luecken et al. 2022; Ronan, Qi, and Naegle 2016)](https://paperpile.com/c/cT6Rz4/vIT8+pg75), and others, there should not be a single implementation, as the selection of method highly depends on the data at hand).

Additionally, we maintain that our analysis in **Figure S8** does not call into question the robustness of our conclusions. While it is true that applying UMAP generally reduces the batch integration score across all methods, Geneformer and scGPT still underperform compared to simpler methods (we now added a **new Table S5**, reproduced below, to improve the presentation of the data). We argue this result actually reinforces the robustness of our conclusions, because it suggests that Geneformer and scGPT underperform across multiple single-cell analysis workflows.

The logic behind the gene expression reconstruction experiments was also unclear. For example, it is unclear why one would expect valid reconstruction without considering the cell embedding, given that gene expression varies by cell type. The cell embedding should be integral to the reconstruction process. Perhaps I misunderstood the full details here.

scGPT generates two types of outputs: GEP (gene expression prediction) and GEPC (gene expression prediction from cell embedding), which provide predictions based on different contexts. The GEP objective enables scGPT to learn cell embeddings and gene expressions from the context of surrounding gene expressions. Conversely, the GEPC objective focuses on predicting gene expressions based on previously learned cell embeddings. Similarly, Geneformer predicts gene rankings to learn gene embeddings and represents cell embeddings as the average of gene embeddings. How well each of scGPT and Geneformer are learning the gene expression space can thus be reflected in the GEP/GEPC and ranking prediction tasks, respectively.

We evaluated both outputs from scGPT and the predictive accuracy of Geneformer to assess their performance in fulfilling their respective pretraining tasks (**Figure 2**). Our analysis confirms that scGPT performs better under the GEPC objective, aligning with the reviewer's intuition, although it only occasionally surpasses the baseline of predicting the mean (**Figures 2D**, **S8**). For Geneformer, the approach of deriving cell embeddings from the average of gene embeddings provides a direct linkage between gene rankings and cell representations.

We discuss the motivation behind investigating the pretraining in the “**Results”** section (lines 100-109), reproduced below.

100 We propose two hypotheses as to why Geneformer and scGPT underperform zero-shot compared to the tested baselines. First, it could be that the masked language model pretraining framework used by both scGPT and Geneformer does not produce useful cell embeddings. Second, it could be that scGPT and Geneformer have failed to learn the pretraining task. To understand this distinction, we evaluated the performance of these models on the gene expression reconstruction pretraining task (for scGPT, the bin value for each gene; for Geneformer, the gene rankings) (Fig. 2, Fig. S9). We find that both models face challenges in reconstructing gene expression (Fig. 2). Without conditioning on its cell embedding, scGPT predicts the mean value of the input bin across all binned values (Fig. 2A). Predictions improve when conditioned on cell embeddings, particularly for higher input values (Fig. 2B). Under its masked language modeling (MLM) objective, Geneformer predicts the most likely gene at a given position. Although there is good agreement across many genes, there are cases in which Geneformer predicts genes absent from the input (Fig. 2C, Fig. S9).

A key motivation for using foundation models in the single-cell space is to learn gene-gene interactions that are cell-type or state-specific and too subtle to detect with simpler models and smaller datasets. Therefore, a thorough analysis of the learned interactions or features compared to simpler models would be a more direct and insightful approach to evaluating the merits of large foundation models.

We agree with the reviewer’s comments that analyzing gene-gene interactions or features from these models would be fascinating and is currently underexplored. However, as we highlighted in our responses above, we consider this out of scope for our current work, which is motivated by assessing the claim advanced by authors of single-cell foundation model papers that these models can capture cell type and state.

Nonetheless, we do think better understanding how single-cell foundation models understand and represent genes would be an important future direction, and we have added a point to this end to the discussion (lines 150-155):

145 ***Another promising avenue for future research on improving these models, closely aligned with current trends in the field, is a more in-depth exploration of how they discern gene-gene interactions. Careful evaluation of this aspect is essential but requires thoughtful consideration and ideally should be paired with targeted in vitro lab experiments. These experiments are crucial for establishing a ground truth, which, in turn, enables an accurate assessment of the models’ true capabilities in unraveling complex biological interactions. A collaborative approach that combines computational evaluation with experimental validation can significantly strengthen and enhance the quality of work in designing these models.***

References

[Ando, D. Michael, Cory Y. McLean, and Marc Berndl. 2017. “Improving Phenotypic Measurements in High-Content Imaging Screens.” *bioRxiv*. bioRxiv. https://doi.org/](http://paperpile.com/b/cT6Rz4/cPd6)[10.1101/161422](http://dx.doi.org/10.1101/161422)[.](http://paperpile.com/b/cT6Rz4/cPd6)

[Cheng, Peng, Cong Mao, Jin Tang, Sen Yang, Yu Cheng, Wuke Wang, Qiuxi Gu, et al. 2024. “Zero-Shot Prediction of Mutation Effects with Multimodal Deep Representation Learning Guides Protein Engineering.” *Cell Research*, July. https://doi.org/](http://paperpile.com/b/cT6Rz4/Qk2n)[10.1038/s41422-024-00989-2](http://dx.doi.org/10.1038/s41422-024-00989-2)[.](http://paperpile.com/b/cT6Rz4/Qk2n)

[Cui, Haotian, Chloe Wang, Hassaan Maan, Kuan Pang, Fengning Luo, Nan Duan, and Bo Wang. 2024. “scGPT: Toward Building a Foundation Model for Single-Cell Multi-Omics Using Generative AI.” *Nature Methods* 21 (8): 1470–80.](http://paperpile.com/b/cT6Rz4/McJn)

[Daudert, Dillon G. 2018. *Exploring the Impact of Pretrained Bidirectional Language Models on Protein Secondary Structure Prediction*.](http://paperpile.com/b/cT6Rz4/Hilx)

[Heumos, Lukas, Anna C. Schaar, Christopher Lance, Anastasia Litinetskaya, Felix Drost, Luke Zappia, Malte D. Lücken, et al. 2023. “Best Practices for Single-Cell Analysis across Modalities.” *Nature Reviews. Genetics* 24 (8): 550–72.](http://paperpile.com/b/cT6Rz4/nkwO)

[Hsu, Chloe, Hunter Nisonoff, Clara Fannjiang, and Jennifer Listgarten. 2022. “Learning Protein Fitness Models from Evolutionary and Assay-Labeled Data.” *Nature Biotechnology* 40 (7): 1114–22.](http://paperpile.com/b/cT6Rz4/DEx4)

[Israel, Uriah, Markus Marks, Rohit Dilip, Qilin Li, Changhua Yu, Emily Laubscher, Shenyi Li, et al. 2024. “A Foundation Model for Cell Segmentation.” *bioRxiv : The Preprint Server for Biology*, March. https://doi.org/](http://paperpile.com/b/cT6Rz4/rTSj)[10.1101/2023.11.17.567630](http://dx.doi.org/10.1101/2023.11.17.567630)[.](http://paperpile.com/b/cT6Rz4/rTSj)

[Luecken, Malte D., M. Büttner, K. Chaichoompu, A. Danese, M. Interlandi, M. F. Mueller, D. C. Strobl, et al. 2022. “Benchmarking Atlas-Level Data Integration in Single-Cell Genomics.” *Nature Methods* 19 (1): 41–50.](http://paperpile.com/b/cT6Rz4/vIT8)

[Lun, Aaron T. L., Karsten Bach, and John C. Marioni. 2016. “Pooling across Cells to Normalize Single-Cell RNA Sequencing Data with Many Zero Counts.” *Genome Biology* 17 (April):75.](http://paperpile.com/b/cT6Rz4/8qq8)

[Luo, Siyuan, Pierre-Luc Germain, Mark D. Robinson, and Ferdinand von Meyenn. 2024. “Benchmarking Computational Methods for Single-Cell Chromatin Data Analysis.” *Genome Biology* 25 (1): 225.](http://paperpile.com/b/cT6Rz4/mH9W)

[Notin, Pascal, Debora S. Marks, Ruben Weitzman, and Yarin Gal. 2023. “ProteinNPT: Improving Protein Property Prediction and Design with Non-Parametric Transformers.” *bioRxiv : The Preprint Server for Biology*, December. https://doi.org/](http://paperpile.com/b/cT6Rz4/Ni5w)[10.1101/2023.12.06.570473](http://dx.doi.org/10.1101/2023.12.06.570473)[.](http://paperpile.com/b/cT6Rz4/Ni5w)

[Pawlowski, Nick, Juan C. Caicedo, Shantanu Singh, Anne E. Carpenter, and Amos Storkey. 2016. “Automating Morphological Profiling with Generic Deep Convolutional Networks.” *bioRxiv*. bioRxiv. https://doi.org/](http://paperpile.com/b/cT6Rz4/NU7S)[10.1101/085118](http://dx.doi.org/10.1101/085118)[.](http://paperpile.com/b/cT6Rz4/NU7S)

[Qiao, Chang, Yunmin Zeng, Quan Meng, Xingye Chen, Haoyu Chen, Tao Jiang, Rongfei Wei, et al. 2024. “Zero-Shot Learning Enables Instant Denoising and Super-Resolution in Optical Fluorescence Microscopy.” *Nature Communications* 15 (1): 4180.](http://paperpile.com/b/cT6Rz4/853N)

[Ronan, Tom, Zhijie Qi, and Kristen M. Naegle. 2016. “Avoiding Common Pitfalls When Clustering Biological Data.” *Science Signaling* 9 (432): re6.](http://paperpile.com/b/cT6Rz4/pg75)

[Slovin, Shaked, Annamaria Carissimo, Francesco Panariello, Antonio Grimaldi, Valentina Bouché, Gennaro Gambardella, and Davide Cacchiarelli. 2021. “Single-Cell RNA Sequencing Analysis: A Step-by-Step Overview.” *Methods in Molecular Biology*  2284:343–65.](http://paperpile.com/b/cT6Rz4/raVd)

[Yu, Lijia, Yue Cao, Jean Y. H. Yang, and Pengyi Yang. 2022. “Benchmarking Clustering Algorithms on Estimating the Number of Cell Types from Single-Cell RNA-Sequencing Data.” *Genome Biology* 23 (1): 49.](http://paperpile.com/b/cT6Rz4/a0ry)

**Second round of review**

**Reviewer 1**

I would like to thank the authors for carefully and thoroughly addressing my comments. Specifically, the aggregation and summarization of the metrics has improved and allows clearer comparison. I understand that some suggestions are out of scope for this paper, but I hope to see future work investigating questions asked by me and the other reviewer.
